# Prevalence and risk factors for loneliness among individuals with diabetes: a systematic review and meta-analysis

**DOI:** 10.1186/s13643-025-02850-y

**Published:** 2025-05-01

**Authors:** Anggi Lukman Wicaksana, Renny Wulan Apriliyasari, Pei-Shan Tsai

**Affiliations:** 1 School of Nursing, College of Nursing, Taipei Medical University, No. 250 Wuxing Street, Xinyi District, Taipei City, 110 Taiwan; 2 Department of Medical Surgical Nursing, Universitas Gadjah Mada, Yogyakarta, Indonesia; 3 Department of Nursing, Institut Teknologi Kesehatan Cendekia Utama Kudus, Kudus, Indonesia; 4 Department of Nursing and Research Center in Nursing Clinical Practice, Wan Fang Hospital, Taipei Medical University, Taipei, Taiwan; 5 Research Center of Sleep Medicine, Taipei Medical University Hospital, Taipei, Taiwan

**Keywords:** Diabetes mellitus, Loneliness, Meta-analysis, Prevalence, Psychological distress, Psychosocial, Risk factors

## Abstract

**Background:**

Loneliness is more pronounced in individuals with diabetes; however, limited studies have investigated loneliness and its risk factors. This study estimated the pooled prevalence of loneliness and identified its risk factors in individuals with diabetes.

**Methods:**

A systematic review and meta-analysis of observational studies was conducted. CINAHL, Cochrane, Embase, PubMed, Scopus, and Web of Science databases were searched from their inception to September 22, 2023. We systematically searched and analyzed 10 studies involving 6036 individuals with diabetes to determine the pooled prevalence of loneliness. Five studies provided information on risk factors. Using a random-effects model, we calculated prevalence rates and odds ratios with 95% confidence intervals.

**Results:**

The overall prevalence of loneliness was 31.1% and severe loneliness was 4.6%. White race, lower education level, middle income, low income, longer diabetes duration, lower cognitive function, living alone, previous loneliness experience, and depression were identified as significant risk factors for loneliness in individuals with diabetes.

**Conclusion:**

Over 30% of individuals with diabetes experience loneliness. Several sociodemographic factors, low cognitive function, and depression are risk factors for loneliness.

**Supplementary Information:**

The online version contains supplementary material available at 10.1186/s13643-025-02850-y.

## Background

Loneliness, characterized by a perceived lack of or a deficit in the quality and/or quantity of social interactions, often leads to psychological distress [1]. This condition has increased the health-care burden and mortality rates among older adults [2, 3]. Moreover, loneliness is a crucial risk factor for type 2 diabetes [4, 5]. Individuals experiencing loneliness have a two-fold higher risk of having type 2 diabetes than those not experiencing loneliness do [4, 6]. Song et al. [7] observed that feeling of loneliness and decreased participation in social activities increase the risk of type 2 diabetes, whereas frequent interactions with family or friends can reduce this risk. Similar to other forms of psychological distresses, loneliness triggers a stress response, increasing vulnerability to physical and mental health problems, and potentially precipitating the onset of diabetes [5, 6, 8]. Additionally, diabetes-related complications can impair physical capabilities, lead to depression, and reduce social relationships [9]. Thus, loneliness and perceived social isolation are more pronounced in individuals with diabetes, even those receiving support from social networks and family, than in those without diabetes [10].

Several meta-analyses have investigated the prevalence of loneliness in the general population [11, 12] and older adults [13–16]. However, limited attention has been given to examining loneliness in individuals with diabetes. Moreover, the few studies addressing this problem have often included small samples [17, 18]. A secondary analysis of a randomized controlled trial involving approximately 5000 individuals with type 2 diabetes highlighted this limitation [19]; the data in that trial were collected from only a single population. Furthermore, few studies have explored risk factors for loneliness among individuals with diabetes [20]. Age, sex, marital status, depression, living arrangements, and an inadequate social network have been identified as risk factors for loneliness among older people [15, 21–23]. However, the risk factors for loneliness among individuals with diabetes remain a topic of debate. Thus, risk factors for loneliness in this population should be identified.

To the best of our knowledge, no study has comprehensively assessed the pooled prevalence of and risk factors for loneliness in individuals with diabetes. Thus, we conducted a systematic review and meta-analysis of the available evidence to determine the prevalence of loneliness and its associated risk factors among individuals with diabetes.

## Methods

### Data sources and searches

The study protocol was previously registered with PROSPERO (CRD42023394369). We conducted a systematic search across six databases, namely CINAHL, Cochrane, Embase, PubMed, Scopus, and Web of Science, from their inception to September 22, 2023 following the Cochrane guideline [24]. This study used Preferred Reporting Items for Systematic Reviews and Meta-Analyses (PRISMA checklist) and the Meta-Analyses of Observational Studies in Epidemiology to report the findings (MOOSE checklist). The following keywords were used during the searches: adult, elderly, diabetes, loneliness, social isolation, prevalence, and risk factor (Supplementary Table 1). In addition, manual searches of references and related articles were performed. All searches were independently conducted by two authors, and disagreements between the two reviewers were resolved through discussion with a third author.

### Study selection

We included studies that 1) reported the prevalence and/or risk factors of loneliness in individuals with diabetes, 2) recruited either adults or older individuals, 3) were observational studies or randomized controlled trials, and 4) were published as full papers in any language. As the research team members were only proficient in English, Chinese, and Indonesian, we used online machine translators (Microsoft Translator, PROMT-online Translation, or Google Translate) to assist us in interpreting and extracting data from papers written in other languages [25]. These tools have acceptable sensitivity and specificity for straightforward tasks [26]. To ensure the comparability of estimates and the representativeness of the population, we excluded studies with a sample size of less than 166. The minimum sample size for inclusion in this study was determined using the formula proposed by Naing et al. [27], with the calculation in the current study based on an estimated loneliness prevalence of 12.3% among individuals with diabetes [28]. Unpublished papers (i.e. preprints, free papers, dissertation, thesis) and conference papers were also excluded.

### Eligibility screening and data extraction

Two authors independently conducted eligibility screening and data extraction. Duplicates were identified and removed using EndNote X9 software (Thompson ISI Research Soft, Philadelphia, PA, US). Titles and abstracts were first screened for eligibility by two authors. Full texts of potentially eligible studies were retrieved for further assessment. The two authors independently assessed these full texts based on the predefined inclusion and exclusion criteria. Any discrepancy was resolved either through discussion between the two authors or, if necessary, consultation with a third author. In cases where the study populations were individuals with diabetes, further details on the prevalence of and risk factors for loneliness in diabetes were obtained through personal communication with the corresponding authors. Furthermore, if studies reported only the mean score of loneliness without prevalence data, the authors were contacted to obtain the complete dataset. Only papers with complete datasets were included in the analysis. Requests for datasets were made exclusively for individuals with diabetes to ensure comparability with other studies, especially when the study population includes not only individuals with diabetes (i.e. general people, older population).

Data were extracted using a self-developed form that included general information (first author’s name, year, country, region as defined by the World Health Organization, and the country’s economic status as indicated by World Bank income levels), study characteristics (study design, sample size, tools used for assessing loneliness, number of scale items, and interpretations), and participants’ characteristics (percentage of women, age, diabetes duration, and type of diabetes). In addition, the form captured data on the prevalence of and the risk factors for loneliness. We considered the following known risk factors of loneliness based on previous research findings: demographic factors (age, sex, ethnicity, marital status, employment status, education level, and the family’s economic status), health-related factors (morbidity, body mass index, diabetes duration, glycosylated hemoglobin (HbA1c) levels, and cognitive function), social factors (living alone, previous experiences of loneliness, and social isolation), and psychological factors (depression) [14, 16, 29].

We observed variations in the methods used to measure loneliness in the included studies. Following the approach used in a previous meta-analysis involving the older population [16], we categorized the methods into two groups: 1) those involving a single-item question and 2) those involving established loneliness scales. Single-item questions are typically used to assess the frequency of loneliness through questions such as “Do you ever feel lonely?” with nominal or ordinal response options, such as “lonely” and “not lonely” or “always lonely,” “often lonely,” “sometimes lonely,” and “never lonely.” When a study dataset included ordinal responses, we followed the conversion protocol used in a previous meta-analysis [16] and categorized responses as “lonely” and “not lonely”. Responses categorized as “lonely” included “always lonely,” “often lonely,” “severely lonely,” “a high degree of loneliness,” “lonely most of the time,” “lonely half of the time,” “a moderate degree of loneliness,” “sometimes lonely,” and any response indicating loneliness from dichotomous options (lonely vs. not lonely). By contrast, responses categorized as “not lonely” included “never lonely,” “rarely lonely,” “seldom lonely,” “a low degree of loneliness,” and “not lonely.” Regarding the established loneliness scales, namely the University of California Los Angeles (UCLA) Loneliness Scale , the De Jong Gierveld Loneliness Scale, and the short version of the Social and Emotional Loneliness Scale, we used well-established cutoffs to differentiate between individuals with diabetes with and without loneliness. Following the categorization used in a previous study [16], the label “severely lonely” was assigned when the dataset provided information about the responses indicated “always lonely,” “often lonely,” “severe lonely,” “a high degree of loneliness,” and “lonely most of the time.”

### Quality assessment

The two authors independently examined the quality of the included studies. Any discrepancies were resolved through discussion between the two authors or, if necessary, further consultation with a third author. We used the risk of bias assessment tool developed by Hoy et al. [30] for observational studies. This tool includes 10 items to evaluate internal validity (items 5–10) and external validity (items 1–4) across four domains; measurement bias, bias related to the analysis, selection bias, and non-response bias. A score of 1 (yes) or 0 (no) is assigned to each item. The final scores are summed, with scores of 9 and 10 indicating a low risk of bias, 7 and 8 indicating a moderate risk of bias, and ≤ 6 indicating a high risk of bias [30]. We investigated the interrater agreement between the two authors by using Cohen’s Kappa test. A Kappa statistic of > 0.90, 0.80–0.90, 0.60–0.79, 0.40–0.59, 0.21–0.39, and 0–0.20 indicates almost perfect agreement, strong agreement, moderate agreement, weak agreement, minimal agreement, and no agreement, respectively [31]. The revised Cochrane Risk of Bias tool was used to assess the quality of randomized controlled trials. It includes five domains and an overall bias judgment, categorized as low risk of bias, some concerns, or high risk of bias [32].

### Data synthesis and analysis

Narrative synthesis was performed to evaluate the characteristics of the studies and participants. Data on the prevalence of loneliness were collected and transformed into event rates, presented as proportions with standard errors and 95% confidence intervals (CI) [16]. Crude summary estimates of prevalence were calculated using Comprehensive Meta-Analysis software version 2 (CMA, Englewood NJ, US) with a random-effects model. If data involving responses indicating severe loneliness, moderate loneliness, and no loneliness were available, they were included in the meta-analysis. Risk factors were identified using odds ratios (ORs) or beta coefficients (*β*), along with *p* values or associated 95% CI. In cases where complete data were not available, correlation coefficients or mean differences from published papers were used, especially if the original authors did not provide additional data upon request. Heterogeneity among studies was assessed using I^2^ and Q statistics, with significant heterogeneity or differences in true effects indicated by *p* < 0.10. Heterogeneity was classified as low (< 25%), moderate (25%–50%), or high (> 50%) [33]. If high heterogeneity was detected, a moderator analysis was performed. General information from papers, study characteristics, and participant characteristics were included as moderators.

A sensitivity analysis was conducted in which we systematically excluded one study at a time to assess the robustness of the prevalence estimates. Publication bias was evaluated using a funnel plot and Egger’s linear regression. Significant publication bias was indicated by a *p* value of < 0.10 [34]. If publication bias was detected, the trim-and-fill approach was applied [35].

### Data and resource availability

All data used in this analysis were gathered from published articles. Additional data were obtained through personal communication with the corresponding or listed authors. All data used in this study are provided in this paper and its supplementary materials.

## Results

### Search results and characteristics

**Fig. 1:**
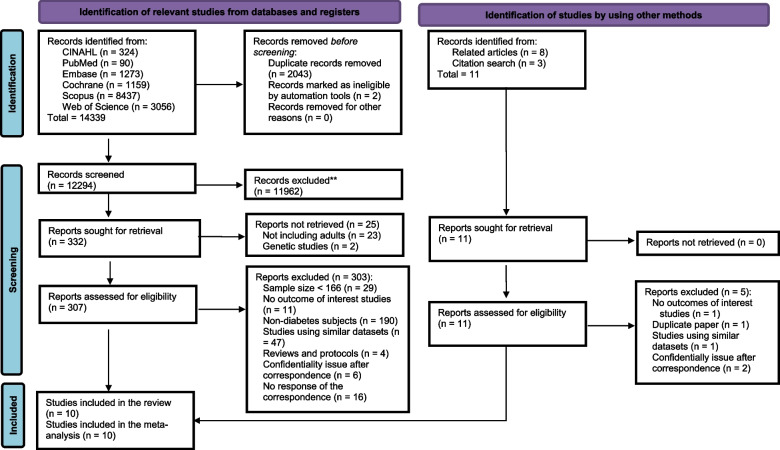
Study flowchart. **If automation tools were used, indicate how many records were excluded by a human and how many were excluded by automation tools

The search of the included databases yielded 14,339 studies. Subsequently, 2043 duplicates were removed. Initial screening led to further exclusion of 11,962 studies. The full text of 307 studies was retrieved and assessed for eligibility. Of these, 303 studies were excluded because they did not meet the inclusion criteria including 190 studies that did not focus on individuals with diabetes. Correspondence was required for several studies; however, we encountered problems such as confidentiality concerns, lack of diabetes-specific data, and nonresponses (Supplementary Table 2). Six additional studies were identified from alternative sources. Finally, 10 papers were included in the meta-analysis (Fig. 1).

Ten studies [5, 17, 20, 28, 36–41] provided data on the prevalence of loneliness in individuals with diabetes (Table 1). All studies used an observational design. Among them, five studies [5, 17, 20, 28, 40] examined risk factors for loneliness. Collectively, these studies included 6036 individuals living with type 1, type 2, or other types of diabetes. The majority of the studies were conducted in European countries (*n* = 4, 40%) and high-income countries (*n* = 7, 70%), with most employing a cohort design (*n* = 4, 40%). The UCLA Loneliness Scale or its revised version (revised UCLA Loneliness Scale) was used to assess loneliness in the majority of the studies (*n* = 6, 60%). Participant characteristics were not clearly reported in some studies (*n* = 4, 40%). Approximately half of the participants were women (mean = 50.03%) with an average age of 64.36 years and an average diabetes duration of 7.26 years.

**Table 1:** Summary of the characteristics of included articles

No.	General information				Study characteristics					Participant characteristics				Outcomes of interest		
	Author/Year	Country	Region	Economic status	Study design	Sample size	Loneliness assessment tools used	No. of scale items	Interpretations	% of Women	Age (year)	DM duration (year)	Type of DM	Prevalence	Risk factors	Types of risk factors
1	Akhter-Khan, 2021 [36]	USA	Americas	HI	Cohort	204	One item from the CES-D	1	Four response categories: No, transient (lonely at visit 1), incident (lonely at visit 2), and persistent loneliness	NI	NI	NI	NI	✔		
2	Chao, 2022 [28]	USA	Americas	HI	Cohort	2829	3i UCLA Loneliness Scale	3	Cutoff for loneliness ≥ 6	63.2	75.6 (6.0)	NI	Type 2 diabetes	✔	✔	Demographic factors (age, sex, and ethnicity), health-related factors (multimorbidity and BMI), and social factors (previous experience of loneliness)
3	Durmus, 2022 [17]	Turkey	Europe	UMI	Cross-sectional	500	20i UCLA Loneliness Scale	20	Higher scores indicated a higher level of loneliness	49.4	49.64 (16.79)	2.36 (1.13)	Type 1 diabetes, Type 2 diabetes	✔	✔	Demographic factors (age, sex, marital status, employment status, education level, and economic status) and health-related factors (duration of diabetes)
4	Hackett, 2020 [5]	The United Kingdom	European	HI	Longitudinal	264	3i Revised UCLA Loneliness Scale	3	The average scores ranged from 1 to 3, with higher scores indicating a higher level of loneliness	45.8	64.62 (8.46)	NI	Type 2 diabetes	✔	✔	Demographic factors (age, sex, and economic status), health-related factors (HbA1c level), social factors (living alone and social isolation), and psychological factor (depression)
5	Kobos, 2021 [20]	Poland	European	HI	Cross-sectional	250	20i Revised UCLA Loneliness Scale	20	Scores of 20–34, 35–49, 50–64, and 65–80 indicate a low, moderate, moderately high, and high degree of loneliness, respectively	46.4	57.93 (17.43)	12.15 (9.55)	Type 1 diabetes, Type 2 diabetes	✔	✔	Demographic factors (age, sex, marital status, and education level)
6	Pengpid, 2023 [37]	Thailand	South-East Asian	UMI	Longitudinal	557	One item from the CES-D	1	Three responses: very rarely or none, often or sometimes, and almost always. Data are categorized as incident (lonely in 2017 but not in 2015) and persistent loneliness (lonely in both 2015 and 2017)	NI	NI	NI	NI	✔		
7	Shibata, 2021 [38]	Japan	Western Pacific	HI	Longitudinal	273	6i DJG Loneliness Scale	6	A score of ≥ 1 indicates the presence of loneliness	NI	NI	NI	NI	✔		
8	Stessman, 2014 [39]	Israel	European	HI	Cohort	253	Single subjective assessment	1	Four responses: never, rarely, often, and very often. The data were classified as dichotomous (not lonely vs. lonely)	NI	NI	NI	NI	✔		
9	Tomida, 2023 [40]	Japan	Western Pacific	HI	Cohort	649	20i Revised UCLA Loneliness Scale v3	20	Cutoff for loneliness ≥ 44	40.1	74 (5.5)	NI	Type 1 diabetes, Type 2 diabetes, other types of diabetes	✔	✔	Demographic factors (age, sex, employment status, and education level), health-related factor (cognitive function), social factor (living alone), and psychological factor (depression)
10	Yousefzadeh, 2021 [41]	Iran	Eastern Mediterranean	LMI	Cross-sectional	257	20i Revised UCLA Loneliness Scale	20	Higher scores indicated a higher level of loneliness	55.3	NI	NI	Type 2 diabetes	✔		

*BMI* Body Mass Index, *CES-D* Center for Epidemiologic Studies Depression Scale, *DJG *De Jong Gierveld, *HbA1c* Glycosylated Hemoglobin, *HI* Higher Income, *i* Item, *LMI* Lower-Middle Income, *N* Number(s), *NI* No Information, *UCLA *University of California Los Angeles, *UMI* Upper-Middle Income, *USA* the United States of America

### Quality assessment

Originally, we planned to use the revised Cochrane Risk of Bias tool [32] to assess the quality of randomized controlled trials. However, as all the included studies were observational in design, quality assessment was conducted using Hoy’s criteria, and the revised Cochrane Risk of Bias tool was deemed inapplicable in this context. The Cohen’s Kappa test, which was performed to examine interrater agreement between the two authors, yielded a value of 0.737 (asymptotic standard error = 0.100, *p* < 0.001). The quality assessment revealed that six studies had a low risk of bias, three had a moderate risk of bias, and one had a high risk of bias. In the detailed assessment of external validity, 80% of the included studies employed a true sampling frame and utilized a random selection process or census while only 60% achieved representativeness of the target population. This indicates a low risk of bias in the selection domain. Additionally, 60% of the studies demonstrated a minimal likelihood of non-response bias, suggesting a low risk in the non-response bias domain of external validity. Regarding internal validity, nearly all criteria related to the measurement bias domain––such as direct data collection, acceptable case definition, use of valid and reliable instruments, and consistent data collection methods––were fulfilled by all studies (100%), except for the use of an appropriate prevalence period, which was achieved by 90% of the studies. All included studies (100%) reported appropriate numerators and denominators for parameters, indicating no bias in the analysis domain of internal validity (Supplementary Table 3).

### Prevalence of loneliness in individuals with diabetes

The pooled prevalence rate of loneliness was estimated using data from the 10 included studies. Of the 6036 individuals with diabetes, 1731 reported loneliness. The pooled prevalence rate of loneliness was 31.10% (95% CI = 21.1% to 43.3%, *p* = 0.003, Table 2). Significant heterogeneity was identified (Q = 597.314, df = 9, *p* < 0.001, I^2^ = 98.49%). Subgroup analyses revealed that study quality (low risk vs. non–low risk, *p* = 0.035) significantly moderated the prevalence of loneliness, with higher-quality (i.e. low risk) studies reporting a slightly but significantly lower prevalence in individuals living with diabetes (Supplementary Table 4).

Only four studies [17, 20, 36, 37] reported severe loneliness, which was observed in 75 of the total 6036 individuals with diabetes. The pooled prevalence rate of severe loneliness was 4.60% (95% CI = 2.2% to 9.5%, *p* < 0.001, Table 2). Substantial heterogeneity among studies was noted (Q = 20.041, df = 3, *p* < 0.001, I^2^ = 85.03%). Moderator analyses indicated that region (European vs. non-European countries, *p* = 0.013), the loneliness assessment tool used (revised or original UCLA Loneliness Scale vs. non-revised or originalUCLA Loneliness Scale, *p* = 0.013) and study quality (low risk vs. non–low risk of bias, *p* = 0.013) were significant factors affecting the prevalence of severe loneliness among individuals with diabetes (Supplementary Table 4).

**Table 2:**
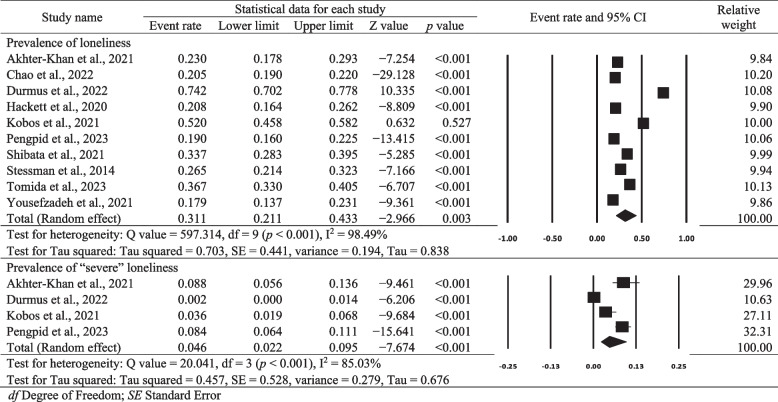
Pooled prevalence of loneliness among individuals with diabetes

### Risk factors for loneliness in diabetes

Five studies presented data on risk factors for loneliness in individuals with diabetes, covering demographic, health-related, social, and psychological factors. White race (OR = 1.410, 95% CI = 1.130–1.740, *p* = 0.002, reference: non-White race), lower education level (pooled OR = 1.620, 95% CI = 1.136–2.309, *p* = 0.008, reference: college and above), middle-level family income (pooled OR = 1.868, 95% CI = 1.194–2.921, *p* = 0.006, reference: high-level family income), low-level family income (pooled OR = 3.212, 95% CI = 1.365–7.557, *p* = 0.008, reference: high-level family income), longer diabetes duration (OR = 1.270, 95% CI = 1.050–1.550, *p* = 0.016), lower cognitive function (OR = 2.586, 95% CI = 1.751–3.781, *p* < 0.001), living alone (pooled OR = 3.359, 95% CI = 1.208–9.342, *p* = 0.020, reference: yes), previous loneliness experience (OR = 7.430, 95% CI = 5.750–9.610, *p* < 0.001, reference: none), and depression (pooled OR = 1.223, 95% CI = 1.173–1.274, *p* < 0.001) were identified as significant risk factors for loneliness among individuals with diabetes (Table 3).

**Table 3:** Risk factors for diabetes-related loneliness

Risk factors	No. of studies	Statistical data			Heterogeneity		
		OR	95% CI	*p* value	I^2^	Q value	*p* value
**Demographic variables**							
Age (year) [5, 17, 20, 28, 40]	5	1.007	0.984–1.031	0.546	72.592	14.594	0.723
Sex (Ref. Male) [5, 17, 20, 28, 40]	5	1.198	0.618–2.324	0.593	93.253	59.282	< 0.001
Ethnicity (Ref. Non-White) [28]	1	1.410	1.130–1.740	0.002			
Marital status (Ref. Married) [17, 20]	2	1.407	0.867–2.281	0.167	48.124	1.928	0.165
Employment status (Ref. Working) [17, 40]	2	1.389	0.977–2.415	0.244	62.742	2.684	0.101
Educational level (Ref. College and above) [17, 40]	2	1.620	1.136–2.309	0.008	0	0.991	0.320
Economic status of the family (Ref. High income) Middle income [5, 17] Low income [5, 17]	2 2	1.868 3.212	1.194–2.921 1.365–7.557	0.006 0.008	0 32.941	0.749 1.491	0.387 0.222
**Health-related variables**							
Multimorbidity (number of morbidities) [28]	1	1.090	0.970–1.230	0.140			
BMI (Ref. < 30 kg m^−2^) [28]	1	1.200	0.980–1.480	0.080			
Diabetes duration (year) [17]	1	1.270	1.050–1.550	0.016			
HbA1c (%) [5]	1	1.015	0.914–1.127	0.779			
Cognitive function (MMSE score) [40]	1	2.586	1.751–3.781	< 0.001			
**Social variables**							
Living alone (Ref. Yes) [5, 40]	2	3.359	1.208–9.342	0.020	83.661	6.120	0.013
Previous experience of loneliness (Ref. None) [28]	1	7.430	5.750–9.610	< 0.001			
Social isolation (Social isolation index) [5]	1	0.947	0.719–1.259	0.707			
**Psychological variable**							
Depression (Depression score) [5, 40]	2	1.223	1.173–1.274	< 0.001	0	9.466	< 0.001

*BMI* Body Mass Index, *HbA1c* Glycosylated Hemoglobin, *MMSE* Mini-Mental State Examination, *OR* Odds Ratio, *Ref* Reference Group

### Sensitivity analysis and publication bias

We performed a meta-analysis on each subset of the studies by removing one study at a time to confirm the robustness of the prevalence result. The leave-one-out meta-analysis resulted in a prevalence rate of loneliness ranging from 26.9% (95% CI = 20.7% – 34.1%) to 32.8% (95% CI = 21.8%–46.2%, Supplementary Table 5). When the analysis excluded a study with a large sample size [28] or one with a high risk of bias [38], the prevalence rate became 32.5% (95% CI = 21.1%–46.4%) or 32.8% (95% CI = 21.8%–46.2%), respectively. These rates were close to that obtained without the removal of any study (rate = 31.10%, 95% CI = 21.1%–43.3%), supporting the robustness of the findings.

A funnel plot used to assess publication bias is presented in Supplementary Fig. 1. Begg and Mazumdar rank correlation analysis yielded Kendall’s tau value of –0.133, z = 0.537, *p* = 0.591, indicating no significant publication bias. Additionally, Egger’s regression analysis revealed an intercept (α) of 4.931 (95% CI = –9.394 to 19.257, df = 8, t = 0.794, *p* = 0.450, indicating the absence of publication bias.

## Discussion

The results of this study revealed that approximately 30% of the individuals with diabetes experienced loneliness. Furthermore, 4.6% of the individuals with diabetes experienced severe loneliness. This study identified demographic, health-related, social, and psychological factors as key determinants of loneliness among individuals with diabetes. Understanding the prevalence of and risk factors for loneliness can help clinicians and stakeholders comprehend the extent of this health problem and mitigate the associated health outcomes and burdens for individuals with diabetes.

Our pooled prevalence analysis revealed that loneliness is extremely prevalent among individuals with diabetes at rates exceeding those reported for older adults (28.5%–28.6%) in previous meta-analyses [13, 14]. Furthermore, longitudinal studies examining the incidence of loneliness among older adults have consistently reported lower rates of 3.8%–29.6%, [15, 23, 42] than those observed for individuals with diabetes in the present study. These results are in line with those of a previous qualitative study on loneliness among American immigrants with diabetes [10]. Collectively, these findings indicate that loneliness is a major health problem in individuals with diabetes.

We found limited data regarding severe loneliness in individuals with diabetes and were only able to include four studies reporting on such loneliness in our analysis. Additionally, few studies have focused on severe loneliness in older adults and the general population. The prevalence rate of severe loneliness reported in previous meta-analyses (7.9%–35%) and a population-based survey (7%) among older people are higher than those observed in individuals with diabetes in the present study [14, 16, 43]. By contrast, the pooled prevalence of severe loneliness was higher in individuals with diabetes in our study than in the general population (1.7%) [12]. These findings suggest an increasing severity of loneliness among older people and less frequent occurrence of such loneliness in the general population. Although the prevalence rates of severe loneliness are low, the consequences of such a state are substantial. Loneliness and social isolation are crucial predictors of mortality among older people [3, 15]. Thus, the problem of severe loneliness warrants attention and should not be neglected.

The pooled prevalence rate of loneliness appears exceptionally high in individuals with diabetes. These findings align with previous studies that reported a high prevalence of loneliness among people living with chronic diseases [18, 44]. Substantial heterogeneity was identified in both the prevalence rates of loneliness and severe loneliness; with I^2^ exceeding 50% [33]. However, sensitivity analyses, conducted by leaving out one study at any time or excluding those with a large sample size or high risk of bias, did not significantly alter the result, suggesting the robustness of our findings. The identified heterogeneity among studies may be attributed to study quality, with studies at low risk of bias showing relatively lower prevalence rates of loneliness compared to those at high risk of bias. In addition, sources of heterogeneity in the prevalence of severe loneliness were related to geographical location where the study was conducted, tools used to assess loneliness, and study quality. Studies from European countries, using the revised or original UCLA Loneliness Scale, and of lower quality indicated relatively lower prevalence rates of severe loneliness. Researchers should consider these moderating variables when assessing the prevalence of loneliness and severe loneliness among individuals with diabetes.

Several demographic and health-related characteristics have been identified as major risk factors for loneliness in individuals with diabetes. Non-Hispanic White individuals with diabetes tend to experience a higher level of loneliness than do those from Native American/Alaskan, American Indian, Asian/Pacific Islander, Hispanic, Black, and mixed ethnic backgrounds [28]. In addition, individuals with diabetes who have a lower education level have a significantly higher risk of loneliness than those with a higher education level from our analysis. This finding is consistent with those of previous analyzing risk factors for loneliness in older individuals [15, 19, 22]. Moreover, individuals with diabetes residing in lower-income households have a threefold higher risk of loneliness than those residing in higher-income households. Huang et al. [23] and Pinquart & Sörensen [29] have reported that unemployed or socioeconomically disadvantaged older people have a higher risk of loneliness. In addition, in this study, a longer duration of living with diabetes was identified as a major risk factor for loneliness. Furthermore, individuals with diabetes who had impaired cognitive function were more likely to experience loneliness. However, caution should be exercised in interpreting findings related to certain risk factors of loneliness, such as ethnicity, diabetes duration, and cognitive function, because the ORs for the association of these factors with loneliness were derived from one single study in our analysis. Additional studies are necessary to obtain pooled estimates of the ORs.

In this study, certain social and psychological factors were identified as significant risk factors for loneliness in individuals with diabetes. Specifically, individuals with diabetes who were living alone were discovered to have a threefold higher risk of experiencing loneliness than those living with family. Moreover, individuals with a history of loneliness were approximately seven times more likely to experience loneliness again compared with those without such a history. These findings are consistent with those of previous studies on older adults that have indicated that living alone considerably increased the risk of loneliness (OR = 1.42–2.78) [15, 22, 23]. Living with family members typically leads to more social interaction than living alone does, and an individual with diabetes having a previous experience of loneliness is predisposed to further episodes of loneliness [28]. In a previous study, British older adults who reported frequent loneliness over the past decade were discovered to be approximately 3.78 times more likely to experience loneliness than those who did not [43]. Individuals with diabetes who experience loneliness have a seven-fold higher risk of experiencing increased loneliness and a four-fold higher risk of experiencing loneliness over the next decade. In addition, in this study, depressive symptoms were identified as a significant risk factor for loneliness among individuals with diabetes. This finding is consistent with our expectations because depression often leads individuals to limit their social interactions and isolate themselves. In the general population, depression was reported to be associated with a two-fold increase in the risk of loneliness [12], a finding consistent with those regarding older adults, for whom increased depressive symptoms have been reported to be associated with a twofold increased risk of loneliness [15, 19, 21]. In the present study, data regarding the effects of previous experiences of loneliness were provided in only a single study. Thus, our conclusions drawn from these data should be interpreted with caution.

Age, sex, HbA1c levels, and social isolation are plausible risk factors for loneliness among individuals with diabetes. However, our analysis did not yield significant results for these factors. By contrast, Pinquart & Sörensen [29] identified a U-shaped association between age and loneliness among older people, with no correlations across most age groups and significant correlations only for individuals aged older than 80 years. In line with this finding, a meta-analysis reported that advanced age was a crucial determinant of loneliness among Chinese older adults [22]. In a recent study, although women with diabetes were anticipated to experience a higher level of loneliness than men with diabetes, sex was not identified as a significant risk factor for loneliness. Previous research has demonstrated that older women typically reported a higher level of loneliness, which substantially contributes to them having an increased risk of experiencing loneliness [15, 29]. However, a longitudinal analysis of older adults in Taiwan identified male sex as a major risk factor for loneliness [23]. Furthermore, the association between HbA1c levels and loneliness among older adults with diabetes has been reported to be nonsignificant [5, 19]. However, a secondary analysis of the Midlife US Survey indicated a significant association between loneliness and HbA1c levels [45]. These conflicting findings indicate that this association remains to be comprehensively elucidated. In addition, inadequate social networks have been identified as a major risk factor for loneliness [15, 21, 22]. Moreover, among older adults, low quality of social interactions was discovered to be more strongly correlated with loneliness than low quantity of social interactions [29]. In the current study, the ORs for the association of HbA1c levels and social isolation with loneliness were derived from only one study. Thus, caution should be exercised in interpreting these findings, especially when pooled ORs from multiple studies could not be obtained.

### Strengths and limitations

The strengths of this meta-analysis include the high quality of the included studies. Most of the studies had a low risk of bias, with robust quality indices. Moreover, this study presented the pooled prevalence of severe loneliness among individuals with diabetes, revealing the substantial adverse effects of loneliness in this population. Sensitivity analyses and an assessment of publication bias support the study findings, confirming the robustness of the study results.

This study reported on the prevalence rate of and risk factors for loneliness with substantial heterogeneity. However, these prevalence rates were derived from only 10 papers. Although we attempted to obtain access to potentially relevant papers through correspondence for more than 3 months, some authors did not respond to our requests (*n* = 16). Thus, the analyses were conducted using the available dataset. Considering the geographical landscape, no studies from Africa, Australia, Central Asia, or Latin America were included in the analysis. An updated meta-analysis on the prevalence of loneliness in diabetes, with contributions from these specific regions or ethnic-specific risk factors, is warranted. As we only included type 1 and type 2 diabetes in the analysis, the findings may primarily apply to this population and may not be generalized to individuals with gestational or other types of diabetes. Further research is needed to explore the prevalence and risk factors of loneliness among individuals with gestational and other types of diabetes. Moreover, data regarding certain risk factors were only available from a single study, making it impossible to calculate the pooled prevalence rates for these factors. Only one study identified HbA1c level as a risk factor for loneliness among individuals with diabetes, and no studies provided evidence on other critical diabetes outcomes on loneliness. Thus, additional studies, especially longitudinal ones, are needed to deepen the understanding of key diabetes outcomes as risk factors for loneliness among individuals with diabetes.

## Conclusions

This meta-analysis examined the pooled prevalence of loneliness, including severe loneliness, among individuals living with diabetes. The study highlights the high prevalence of loneliness and severe loneliness experienced by this population, suggesting a need for early detection and tailored interventions to reduce and prevent loneliness. Significant risk factors included White race, lower education level, lower income, longer diabetes duration, lower cognitive function, living alone, previous loneliness experience, and depression. Individuals with diabetes who have these risk factors should received additional support to help mitigate loneliness. However, certain risk factors, such as ethnicity, diabetes duration, cognitive function, and previous experience of loneliness, should be interpreted with caution due to the odds ratios for these factors were each drawn from one single study. Healthcare providers working with individuals with diabetes should consider these risk factors as important indicators in their efforts to prevent or alleviate loneliness.

## Supplementary Information

Supplementary Material 1.

Supplementary Material 2.

## Data Availability

All raw data required to produce the above findings are available upon request.
