# Supplementary material for: Prevalence and risk factors for loneliness among individuals with diabetes: a systematic review and meta-analysis
Source: Syst Rev. 2025 May 1;14:96. doi: 10.1186/s13643-025-02850-y (PMC12044816; doi:10.1186/s13643-025-02850-y)
Supplement: Supplementary file 1 — Supplementary Material 1. [file 13643_2025_2850_MOESM1_ESM.doc]

**Appendix A**

**Supplementary Table 1.** Searching Strategy

|  | **PubMed**  **(90)** | **Embase**  **(1273)** | **CINAHL**  **(324)** | **Scopus**  **(8437)** | **Web of Science**  **(3056)** | **Cochrane Library**  **(1159)** |
| --- | --- | --- | --- | --- | --- | --- |
| Population 1/ P1: Age | #1 ((((("adult"[MeSH Terms]) OR ("adults"[All Fields])) OR ("aged"[MeSH Terms])) OR ("elderly"[All Fields])) OR ("older people"[All Fields])) OR ("geriatrics"[MeSH Terms]) | #1 'adult'/exp OR 'adult':ab,ti  #2 'adults'/exp OR 'adults':ab,ti  #3 'aged'/exp OR 'aged':ab,ti  #4 'elderly'/exp OR 'elderly':ab,ti  #5 'older people'/exp OR 'older people':ab,ti  #6 'geriatric'/exp OR 'geriatric':ab,ti | TI ("adult" OR "adults" OR "aged" OR "elderly" OR "older people" OR "geriatric")  AND  AB ("adult" OR "adults" OR "aged" OR "elderly" OR "older people" OR "geriatric") | TITLE-ABS-KEY ("adult" OR "adults" OR "aged" OR "elderly" OR "older people" OR "geriatric") | #1 TS=("adult" OR "adults" OR "aged" OR "elderly" OR "older people" OR "geriatric") | ("adult" OR "adults" OR "aged" OR "elderly" OR "older people" OR "geriatric"):ti,ab,kw |
| Population 2/ P2: Patient | #2 (("diabetes"[All Fields]) OR ("diabetes mellitus"[MeSH Terms])) OR ("dm"[All Fields]) | #1 'diabetes'/exp OR 'diabetes':ab,ti  #2 'diabetes mellitus'/exp OR 'diabetes mellitus':ab,ti  #3 'dm'/exp OR 'dm':ab,ti | TI ("diabetes" OR "diabetes mellitus" OR "dm")  AND  AB ("diabetes" OR "diabetes mellitus" OR "dm") | TITLE-ABS-KEY ("diabetes" OR "diabetes mellitus" OR "dm") | #2 TS=("diabetes" OR "diabetes mellitus" OR "dm") | ("diabetes" OR "diabetes mellitus" OR "dm"):ti,ab,kw |
| Interest/I: Loneliness | #3 ((("lonely"[All Fields]) OR ("loneliness"[MeSH Terms])) OR ("alone"[All Fields]) OR ("social isolation"[MeSH Terms])) | #1 'lonely'/exp OR 'lonely':ab,ti  #2 'loneliness'/exp OR 'loneliness':ab,ti  #3 'alone'/exp OR 'alone':ab,ti  #4 'social isolation'/exp OR ' social isolation':ab,ti | TI ("lonely" OR "loneliness" OR "alone" OR "social isolation")  AND  AB ("lonely" OR "loneliness" OR "alone" OR "social isolation") | TITLE-ABS-KEY ("lonely" OR "loneliness" OR "alone" OR "social isolation") | #3 TS=("lonely" OR "loneliness" OR "alone" OR "social isolation") | ("lonely" OR "loneliness" OR "alone" OR "social isolation"):ti,ab,kw |
| Outcome 1/ O1: Prevalence | #4 (((("prevalence"[ MeSH Terms]) OR ("epidemiology"[ MeSH Terms])) OR ("sentinel surveillance"[MeSH Terms])) OR ("surveillance"[All Fields])) OR ("event rates"[All Fields]) | #1 ' prevalence'/exp OR ' prevalence':ab,ti  #2 'epidemiology'/exp OR 'epidemiology':ab,ti  #3 'sentinel surveillance'/exp OR 'sentinel surveillance':ab,ti  #4 'surveillance'/exp OR 'surveillance':ab,ti  #5 'event rates'/exp OR 'event rates':ab,ti | TI ("prevalence" OR "epidemiology" OR "sentinel surveillance" OR "surveillance" OR "event rates")  AND  AB ("prevalence" OR "epidemiology" OR "sentinel surveillance" OR "surveillance" OR "event rates") | TITLE-ABS-KEY ("prevalence" OR "epidemiology" OR "sentinel surveillance" OR "surveillance" OR "event rates") | #4 TS=("prevalence" OR "epidemiology" OR "sentinel surveillance" OR "surveillance" OR "event rates") | ("prevalence" OR "epidemiology" OR "sentinel surveillance" OR "surveillance" OR "event rates"):ti,ab,kw |
| Outcome 2/ O2: Risk factors | #5 ("risk factors"[MeSH Terms]) OR ("risk factor"[All Fields]) OR ("determinant factor"[All Fields]) OR ("associated factor"[All Fields]) OR ("predictor"[All Fields]) | #1 'risk factors'/exp OR 'risk factors':ab,ti  #2 'risk factor'/exp OR 'risk factor':ab,ti  #3 'determinant factor'/exp OR 'determinant factor':ab,ti  #4 'associated factor'/exp OR 'associated factor':ab,ti  #5 'predictor'/exp OR 'predictor':ab,ti | TI ("risk factors" OR "risk factor" OR "determinant factor" OR "associated factor" OR "predictor")  AND  AB ("risk factors" OR "risk factor" OR "determinant factor" OR "associated factor" OR "predictor") | TITLE-ABS-KEY ("risk factors" OR "risk factor" OR "determinant factor" OR "associated factor" OR "predictor") | #5 TS=("risk factors" OR "risk factor" OR "determinant factor" OR "associated factor" OR "predictor") | ("risk factors" OR "risk factor" OR "determinant factor" OR "associated factor" OR "predictor"):ti,ab,kw |
| Final search | #6 (#1 OR #2)  #7 (#6 AND #3 AND #4 AND #5) | (P1 OR P2) AND I AND O1 AND O2 | (SP1 OR SP2) AND SI AND SO1 AND SO2 |  | #6 (#1 OR #2)  #7 (#6 AND #3 AND #4 AND #5) |  |
| Limits applied | Free full text, Clinical trial, Controlled clinical trial, Observational study, Randomized controlled trial, Human, Adult (19+) | Article, Adult (18-64 OR aged 65+), Humans, Diabetes mellitus | Human, Adult (19-44 years), Middle age (45-64 years), Aged (65+ years) | Medicine, Nursing, Psychology, Social sciences, Article, Journal paper, Final publication | Article type,  Not proceeding paper or book chapters | Trial |

**Appendix B**

**Supplementary Table 2.** List of potential papers for inclusion but then it excluded after correspondences

| **No** | **Reason for exclusion** | **Author, year** | **Title** |
| --- | --- | --- | --- |
| 1 | Confidential issue | Foti, 2020 | Loneliness, cardiovascular disease, and diabetes prevalence in the Hispanic Community Health Study/Study of Latinos Sociocultural Ancillary Study |
| 2 |  | Freak-Poli, 2022 | Loneliness, Not Social Support, Is Associated with Cognitive Decline and Dementia Across Two Longitudinal Population-Based Cohorts |
| 3 |  | Kucharska-Newton, 2021 | Loneliness and its predictors among older adults prior to and during the COVID-19 pandemic: cross-sectional and longitudinal survey ndings from participants of the Atherosclerosis Risk in Communities (ARIC) Study cohort in the USA |
| 4 |  | Powell, 2021 | Bad company: Loneliness longitudinally predicts the symptom cluster of pain, fatigue, and depression in older adults |
| 5 |  | Richard, 2017 | Loneliness is adversely associated with physical and mental health and lifestyle factors: Results from a Swiss national survey |
| 6 |  | Sutin, 2022 | Loneliness and risk of all cause Alzheimers vascular and frontotemporal dementia: A prospective study of 492,322 individuals over 15 years. |
| 7 |  | Takahashi, 2023 | Social Isolation/Loneliness andTooth Loss in Community- Dwelling Older Adults: The Sukagawa Study |
| 8 |  | Yu, 2020 | Social Isolation and Loneliness as Risk Factors for Grip Strength Decline Among Older Women and Men in China |
| 9 | No response | Acikgoz, 2022 | Relationship Between Loneliness, Physical Activity, and Depressive Symptoms among Older Adults: A Cross-Sectional Study Conducted During The Fourth Wave of The Covid-19 Pandemic in Turkey |
| 10 |  | Allen, 2022 | The public health impact of loneliness during the COVID-19 pandemic |
| 11 |  | Altintas, 2023 | Relationship between frailty and loneliness among community-dwelling Turkish older people |
| 12 |  | Ayalon, 2011 | The relationship between loneliness and passive death wishes in the second half of life |
| 13 |  | Bekhet, 2012 | Mental Health of Elders in Retirement Communities: Is Loneliness a Key Factor? |
| 14 |  | Carrasco, 2022 | Loneliness in the Elderly: Association with Health Variables, Pain, and Cognitive Performance. A Population-based Study |
| 15 |  | Cheng, 2015 | Disparities in prevalence and risk indicators of loneliness between rural empty nest and non-empty nest older adults in Chizhou, China |
| 16 |  | Jia, 2020 | The association between sleep quality and loneliness in rural older individuals: a cross- sectional study in Shandong Province, China |
| 17 |  | Jiang, 2022 | Relationship between hearing loss and depression symptoms among older adults in China: The mediating role of social isolation and loneliness |
| 18 |  | Julsing, 2016 | Loneliness and All-Cause, Cardiovascular and Non-Cardiovascular Mortality in Older Men: The Zutphen Elderly Study |
| 19 |  | Kirkland, 2023 | Increased prevalence of loneliness and associated risk factors during the COVID-19 pandemic: ndings from the Canadian Longitudinal Study on Aging (CLSA) |
| 20 |  | Montejo, 2019 | Memory complaints: mental health, diseases, pain and loneliness. A population study in the city of Madrid |
| 21 |  | Murayama, 2023 | Changes in social isolation and loneliness prevalence during the COVID-19 pandemic in Japan: The JACSIS 2020–2021 study |
| 22 |  | O’Shea, 2023 | Loneliness Among US Adults Aged ≥55 Early in the COVID-19 Pandemic: Findings From the COVID-19 Coping Study |
| 23 |  | Smith, 2021 | Is loneliness associated with mild cognitive impairment in low‐ and middle‐income countries? |
| 24 |  | Zhang, 2018 | Loneliness and Health Service Utilization among the Rural Elderly in Shandong, China: A Cross-Sectional Study |

**Appendix C**

Supplementary Table 3. Risk of Bias Assessment of The Included Randomized Controlled Trial Studies

| **Study** | **External validity** | | | | **Internal validity** | | | | | | **Total** | **Overall risk of bias** |
| --- | --- | --- | --- | --- | --- | --- | --- | --- | --- | --- | --- | --- |
|  | **Representativeness** | **Sampling frame** | **Random selection/ census** | **Non-response** | **Data collection** | **Case definition** | **Instrument** | **Same mode** | **Prevalence period** | **Numerator/ denominator** |  |  |
| Akhter-Khan et al., 2021 | 1 | 1 | 1 | 1 | 1 | 1 | 1 | 1 | 1 | 1 | 10 | Low |
| Chao et al., 2022 | 1 | 1 | 1 | 0 | 1 | 1 | 1 | 1 | 1 | 1 | 9 | Low |
| Durmus et al., 2022 | 0 | 1 | 0 | 1 | 1 | 1 | 1 | 1 | 1 | 1 | 8 | Moderate |
| Hacket et al., 2020 | 1 | 1 | 1 | 1 | 1 | 1 | 1 | 1 | 1 | 1 | 10 | Low |
| Kobos et al., 2021 | 0 | 0 | 1 | 1 | 1 | 1 | 1 | 1 | 1 | 1 | 8 | Moderate |
| Pengpid et al., 2023 | 1 | 1 | 1 | 0 | 1 | 1 | 1 | 1 | 1 | 1 | 9 | Low |
| Shibata et al., 2021 | 0 | 1 | 1 | 0 | 1 | 1 | 1 | 1 | 1 | 1 | 8 | Moderate |
| Stessman et al., 2014 | 1 | 1 | 1 | 1 | 1 | 1 | 1 | 1 | 1 | 1 | 10 | Low |
| Tomida et al., 2023 | 1 | 1 | 1 | 0 | 1 | 1 | 1 | 1 | 1 | 1 | 9 | Low |
| Yousefzadeh et al., 2021 | 0 | 0 | 0 | 1 | 1 | 1 | 1 | 1 | 0 | 1 | 6 | High |

**Appendix D**

**Supplementary Table 4. The Summary of Moderator Analysis**

| **Group** | **Number of study** | **Sub-group analysis** | | | | |
| --- | --- | --- | --- | --- | --- | --- |
|  |  | **Event rate** | **95% Confidence Interval** | **I^2^** | **Q-value** | ***p*-value** |
| **Loneliness** |  |  |  |  |  |  |
| Region | 10 | 0.324 | 0.176-0.519 | 98.493 | 3.456 | 0.063 |
| European | 4 | 0.427 | 0.274-0.594 | 98.730 |  |  |
| Non-European | 6 | 0.245 | 0.158-0.361 | 95.051 |  |  |
| Economic status of the country | 10 | 0.311 | 0.210-0.434 | 98.493 | 0.157 | 0.692 |
| High income | 7 | 0.296 | 0.183-0.442 | 96.557 |  |  |
| Non-high income | 3 | 0.347 | 0.168-0.583 | 99.420 |  |  |
| Study design | 10 | 0.311 | 0.211-0.433 | 98.493 | 0.525 | 0.469 |
| Cohort | 4 | 0.263 | 0.135-0.449 | 96.035 |  |  |
| Non-cohort | 6 | 0.346 | 0.212-0.510 | 98.753 |  |  |
| Loneliness assessment tool | 10 | 0.311 | 0.205-0.441 | 98.493 | 0.7 | 0.403 |
| R/UCLA LS | 6 | 0.354 | 0.211-0.530 | 99.110 |  |  |
| Non-R/UCLA LS | 4 | 0.252 | 0.122-0.449 | 86.345 |  |  |
| Risk of bias | 10 | 0.327 | 0.168-0.539 | 98.493 | 4.443 | 0.035 |
| Low risk | 6 | 0.240 | 0.157-0.349 | 94.004 |  |  |
| Non-low risk | 4 | 0.437 | 0.289-0.597 | 98.652 |  |  |
| **“Severe” loneliness** |  |  |  |  |  |  |
| Region | 4 | 0.043 | 0.010-0.172 | 85.030 | 6.127 | 0.013 |
| European | 2 | 0.019 | 0.007-0.052 | 86.941 |  |  |
| Non-European | 2 | 0.086 | 0.044-0.161 | 0 |  |  |
| Economic status | 4 | 0.037 | 0.011-0.116 | 85.030 | 0.625 | 0.429 |
| High income | 2 | 0.057 | 0.012-0.238 | 80.567 |  |  |
| Non-high income | 2 | 0.022 | 0.004-0.121 | 93.005 |  |  |
| Study design | 4 | 0.039 | 0.013-0.109 | 85.030 | 0.937 | 0.333 |
| Cohort | 1 | 0.088 | 0.012-0.436 | 0 |  |  |
| Non-cohort | 3 | 0.028 | 0.008-0.096 | 89.544 |  |  |
| Loneliness assessment tool | 4 | 0.043 | 0.010-0.172 | 85.030 | 6.127 | 0.013 |
| R/UCLA LS | 2 | 0.019 | 0.007-0.052 | 86.941 |  |  |
| Non-R/UCLA LS | 2 | 0.086 | 0.044-0.161 | 0 |  |  |
| Risk of bias | 4 | 0.043 | 0.010-0.172 | 85.030 | 6.127 | 0.013 |
| Low risk | 2 | 0.086 | 0.044-0.161 | 0 |  |  |
| Non-low risk | 2 | 0.019 | 0.007-0.052 | 86.941 |  |  |

*Notes*: R/UCLA LS = Revised version or University of California Los Angeles Loneliness Scale

**Appendix E**

**Supplementary Table 5. Sensitivity Analyses of The Interested Outcomes**

| **Study name** | | **Prevalence rate (%)** | **95% Confidence interval (%)** | ***p*** |
| --- | --- | --- | --- | --- |
| 1 | Removed Akhter-Khan et al., 2021 | 32.1 | 21.1 – 45.4 | 0.009 |
| 2 | Removed Chao et al., 2022 | 32.5 | 21.1 – 46.4 | 0.015 |
| 3 | Removed Durmus et al., 2022 | 26.9 | 20.7 – 34.1 | <0.001 |
| 4 | Removed Hacket et al., 2020 | 32.4 | 21.4 – 45.8 | 0.011 |
| 5 | Removed Kobos et al., 2021 | 29.1 | 19.1 – 41.5 | 0.001 |
| 6 | Removed Pengpid et al., 2023 | 32.7 | 21.4 – 46.4 | 0.014 |
| 7 | Removed Shibata et al., 2021 | 30.8 | 20.0 – 44.3 | 0.006 |
| 8 | Removed Stessman et al., 2014 | 31.6 | 20.7 – 45.1 | 0.009 |
| 9 | Removed Tomida et al., 2023 | 30.5 | 19.3 – 44.6 | 0.008 |
| 10 | Removed Yousefzadeh et al., 2021 | 32.8 | 21.8 – 46.2 | 0.013 |

**Appendix F**

**Supplementary Figure 1.** Publication bias
